# The Clinical Septet of Van Wyk–Grumbach Syndrome: A Case Series from a Tertiary Care Centre in Kalyana Karnataka, India

**DOI:** 10.17925/EE.2023.19.1.98

**Published:** 2023-02-06

**Authors:** Swaraj Waddankeri, Meenakshi Waddankeri, Shrikant Waddankeri, Kshitij Arora

**Affiliations:** Mahadevappa Rampure Medical College, Kalaburagi, Karnataka, India

**Keywords:** Levothyroxine, precocious puberty, primary hypothyroidism, Van Wyk–Grumbach syndrome

## Abstract

Van Wyk–Grumbach syndrome is a rare, female juvenile hypothyroidism disorder that is characterized by precocious puberty with clinical, radiological and hormonal pathologies. We present a case series of three patients with this unusual condition who were evaluated and followed up over a 3-year period between January 2017 and June 2020. All three patients presented with short stature (<3rd centile), low weight (<3rd centile), absence of goitre, no axillary or pubic hair, delayed bone age by more than 2 years, elevated thyroid-stimulating hormone with low T3 and T4 (primary hypothyroidism), and raised follicle-stimulating hormone with pre-pubertal levels of luteinizing hormone. Abdominal ultrasonography showed bilateral multi-cystic ovaries in two patients and a right-sided bulky ovary in the third patient. One of the patients also had a pituitary ‘macroadenoma’. All the patients were successfully managed with levothyroxine. We discuss the pathophysiological mechanisms with a brief literature review.

Hypothyroidism usually presents with subtle signs and symptoms. Rarely, longstanding juvenile hypothyroidism can manifest as a syndromic diagnosis of Van Wyk–Grumbach syndrome (VWGS).^1^ VWGS presents as early menarche, thelarche, galactorrhoea, delayed bone ageing and multi-cystic ovaries, along with long-standing primary hypothyroidism.^2^ Reversal to the prepubertal state is typically seen following the appropriate treatment with levothyroxine.

The present case series describes three patients with VWGS referred for evaluation of precocious puberty to the Division of Diabetes and Endocrinology, Department of Medicine, Basaweshwar Teaching and General Hospital attached to Mahadevappa Rampure Medical College, Kalaburagi, Kalyana Karnataka, India, between January 2017 and June 2020.

Two patients presented with per vaginal (PV) bleeding as the main complaint, whereas the third patient presented with thelarche and PV bleeding. All had short stature (height <3rd centile), low weight (<3rd centile), absence of goitre, no axillary or pubic hair, delay of bone age by more than 2 years, and high levels of thyroid-stimulating hormone (TSH) with low T3 and T4 at diagnosis. Prepubertal levels of luteinizing hormone (LH) with high levels of follicle-stimulating hormone (FSH) were seen in all patients. Ultrasonography of the abdomen showed bilateral multi-cystic ovaries in two patients, while the third patient had a right-sided bulky ovary. One of the patients also had a pituitary ‘macroadenoma’ as visualized by magnetic resonance imaging (MRI).

## Case 1

**Figure 1:**
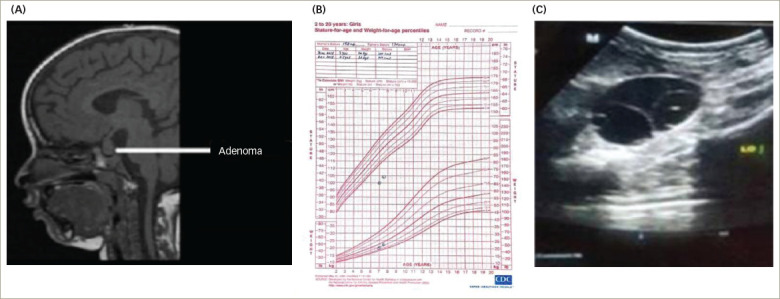
Endocrine Work-up Case 1. A: Pituitary MRI showing thyrotrope hyperplasia mimicking a pituitary adenoma; B: Growth chart; C: Abdominal ultrasound showing polycystic ovaries

A 7-year-old girl was referred to our institution for evaluation of precocious puberty. The parents reported that the child had intermittent vaginal bleeding associated with pain in the abdomen for the past 7 days. Her previous medical team had ordered a pituitary MRI (*Figure 1A*) and performed a work-up for precocious puberty. On further questioning, the parents said that the child was lethargic and constipated. The parents were also concerned about retarded growth, as she was the shortest girl in her class. She was born of a non-consanguineous marriage, at term by normal vaginal delivery. Perinatal transition, developmental milestones and school performance were normal until the age of 6 years. On examination, the patient had facial puffiness along with brittle hair and dry scaly skin. Her height was 100.0 cm (below 3rd centile, according to the standard height for age growth chart) and she weighed 20.0 kg (*Figure 1B*), with a body mass index (BMI) of 20.0 kg/m^2^. The upper/lower segment height ratio was 1.41, and the mid-parental height was 157.5 cm. Sexual maturity grading by Tanner stage was A1B3P1 (A = axillary hair growth, B = breast development, P = pubic hair growth). The remaining systemic examination was unremarkable. *Table 1* and *Table 2* summarize the clinical presentation and hormonal features of Case 1 at diagnosis. Ultrasound of the abdomen and pelvis showed multiple cysts in both the ovaries (*Figure 1C*), and MRI of the pituitary revealed macroadenoma (*Figure 1A*). X-ray of the left hand and wrist suggested a bone age of 5 years using the Greulich and Pyle method.

*MRI = magnetic resonance imaging.*

The patient was initiated on levothyroxine at a dose of 1.5 µg/kg body weight. The patient improved gradually with regular treatment, and at 6 months’ follow-up, the skin texture had improved. PV bleeding stopped 2 months after starting levothyroxine treatment. The parents refused for the child to be photographed, and the patient was subsequently lost to follow-up.

## Case 2

**Figure 2:**
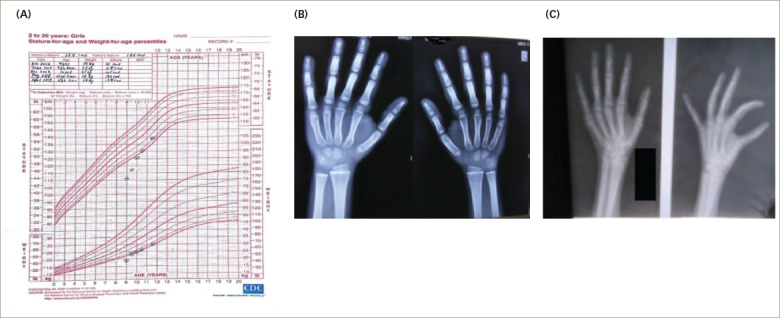
Basic endocrine work-up Case 2. A: Growth chart; B: X-ray at diagnosis; C: X-ray at 12 months post-treatment

A girl aged 7 years and 6 months was brought to our institution by her parents, as she had experienced intermittent vaginal bleeding since the age of 3 months. On detailed questioning, the parents revealed she had a poor scholastic performance over the past year, with her school grades gradually falling. She was born of a non-consanguineous marriage at full term by normal vaginal delivery. The child had cried immediately after birth and had no history of prolonged jaundice, intensive care unit (ICU) stay or convulsions. The perinatal transition and developmental milestones were normal. On examination, she was found to have a hoarse voice, periorbital puffiness, constipation and dry itchy skin. The patient’s height was recorded as 111.0 cm (below 3rd centile) (*Figure 2A*) and she weighed 19.0 kg with a BMI of 15.4 kg/m^2^. The upper/lower height segment ratio was 1.34. The mid-parental height was 149.5 cm. Sexual maturity grading by Tanner stage was A1B2P1 at diagnosis. *Table 1* and *Table 2* summarize the clinical presentation and hormonal features of Case 2 at diagnosis. There was no goitre, and the systemic examination was otherwise unremarkable. The bone age at diagnosis was 2.5 years (Greulich and Pyle method) (*Figure 2B*). An ultrasound of the abdomen and pelvis revealed a bulky right ovary but there were no cysts in the ovaries. The patient was started on levothyroxine at 1.5 µg/kg body weight, and the patient presented for regular follow-up over the next 2 years. PV bleeding ceased after 2 months of treatment, and the patient’s scholastic performance improved. The periorbital swelling and dry skin improved gradually. Bone age of the patient improved with levothyroxine treatment and nutritional and vitamin D supplementation (*Figure 2C*). The patient had a normal menarche at the age of 11 years, and she is doing well with no current illness.

## Case 3

**Figure 3:**
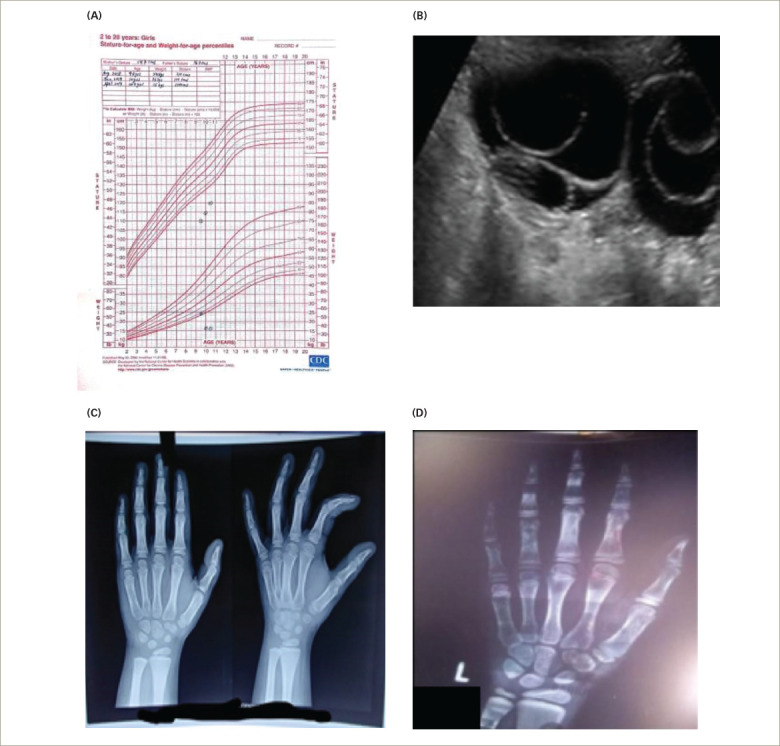
Basic endocrine work-up Case 3. A: Growth chart; B: Abdominal ultrasound showing a polycystic ovary; C: X-ray at diagnosis; D: X-ray at 12 months post-treatment

A girl aged 9 years and 1 month was referred to our institution with a history of inability to gain height, lethargy, weight loss and constipation for 6 months. Her parents came in for a consultation because of PV bleeding over the past 3 days. The child was born of a non-consanguineous marriage. The birth was a full-term, normal vaginal delivery, with no antenatal or post-natal events. The child had cried immediately after birth with no history of recurrent jaundice or ICU stay. Perinatal transition, developmental milestones and school performance were normal. Her height was recorded as 110.0 cm (below 3rd centile) (*Figure 3A*), and she weighed 24.0 kg with a of BMI of 19.8 kg/m^2^. The upper/lower height segment ratio was 1.53, and the mid-parental height was 150.5 cm. Her sexual maturity grading by Tanner stage was A1B2P1 at diagnosis. There was no goitre, and the patient’s systemic examination was unremarkable. The mother’s age at menarche was 12 years. *Table 1* and *Table 2* summarize the clinical presentation and hormonal features of Case 3 at diagnosis. The bone age calculated with the Greulich and Pyle method using an X-ray of the left hand and wrist at presentation was 6.5 years (*Figure 3C*). An ultrasound of the abdomen and pelvis revealed multiple large bilateral ovarian cysts (*Figure 3B*). The child was started on levothyroxine treatment based on weight, and was also given other nutritional supplementation such as calcium and vitamin D. The patient improved clinically, with cessation of bleeding and improvement in skin texture and reduced hair loss over the following 6 weeks. One interesting aspect of the case related to scholastic performance; her scholastic performance was good before initiating therapy with levothyroxine, but contrary to usual norms, her academic performance declined post-treatment, with reports of a poor attention span and hyperactivity. Although, her bone age improved with levothyroxine treatment and nutritional and vitamin D supplementation (*Figure 3D*).

## Discussion

The unusual clinical spectrum of premature menarche, thelarche, delayed bone growth, multi-cystic ovaries and galactorrhoea, resulting from long-standing primary hypothyroidism, was first described by Van Wyk and Grumbach in 1960. Now termed VWGS, the characteristic clinical presentation is a discordant combination of precocious puberty and delayed growth.^3^ The pathogenesis of VWGS involves complex hormonal interactions, with various hypotheses suggested to explain the pathogenesis. The most widely accepted theory is that the high levels of TSH, seen in profound hypothyroidism, interact with the FSH receptors. Gonadotropins and TSH share the same α-subunit but the β-subunit is unique to each hormone. At very high levels, TSH can act as a competitive inhibitor of FSH by interacting with the α-subunit receptors of FSH, stimulating adenylate cyclase activity.^4^ The raised levels of TSH acting via the FSH receptors (‘specificity spill over’) induces FSH-like effects on the gonads, leading to uterine enlargement with bleeding, multi-cystic ovaries and breast enlargement in girls.^5^ In addition, FSH secretion is stimulated by persistently high thyrotropin-releasing hormone (TRH) levels resulting from longstanding profound primary hypothyroidism. Patients with VWGS have a higher upper limit of the age-appropriate levels of FSH this aligns with our patients, all of whom had high FSH levels. Due to the absence of stimulation of adrenarche, axillary and pubic hair growth does not occur. This explains the absence of concomitant pubertal development in patients with VWGS.

**Table 1:** Summary of clinical presentation at diagnosis

Parameter	Case 1	Case 2	Case 3
Age	7 years	7 years and 6 months	9 years and 1 month
Other symptoms	Lethargy Dry skin Constipation	Periorbital puffiness Dry skin Constipation Hoarse voice Poor scholastic performance	Lethargy Weight loss Constipation
Bleeding PV	Yes	Yes	Yes
Thelarche	Yes	No	No
Mother age of menarche (years)	13	12	12
Family history of thyroid illness	No	Yes: Mother and two older siblings, diagnosed with TSH >100.0 mIU/mL	No
Short stature	Yes	Yes	Yes
Tanner stage	A1B3P1	A1B2P1	A1B2P1
Height (cm)	100.0	111.0	110.0
Weight (kg)	20.0	19.0	24.0
BMI (kg/m^2^)	20.0	15.4	19.8
Height centile	<3rd centile	<3rd centile	<3rd centile
Z-score	-4.7	-4.1	-4.4
Goitre (WHO)	Zero	Zero	Zero
Mother height (cm)	158.0	154.0	147.0
Father height (cm)	170.0	166.0	167.0
Mid-parental height (cm)	157.5	149.5	150.5
Natural menarche (years)	–*	11	12

*Unknown as the patient was lost to follow-up.

*BMI = body mass index; PV = per vaginal; WHO = World Health Organization.*

**Table 2:** Summary of hormonal features at diagnosis

Test (normal reference range)	Case 1	Case 2	Case 3
FSH (0.3–10 mIU/mL)	7.3	7.3	12.2
LH (1.5–12 mIU/mL)	<0.1	<0.1	<0.1
Prolactin (5–25 ng/mL)	61.7	64.3	56.4
Oestradiol (<10 pg/mL)	64.0	72.0	67.0
Triiodothyronine (T3) (1.0–2.5 ng/dL)	0.4	0.2	<0.4
Tetraiodothyronine (T4) (4–15 µg/dL)	0.8	0.5	0.7
TSH (0.3–4 mIU/mL)	>100.0	>100.0	>100.0

*FSH = follicle-stimulating hormone; LH = luteinizing hormone; TSH = thyroid-stimulating hormone.*

The incongruous relation between FSH and LH in this syndrome can be explained by the role of prolactin. Hyperprolactinaemia occurs due to unopposed prolactin secretion secondary to elevated TRH levels. Prolactin increases the sensitivity of the ovaries to gonadotropins, while slowing down gonadotropin-releasing hormone pulse frequency.^5,6^ The prolactin was elevated in all three patients in this series, supporting the prolactin theory. Durbin and colleagues reported VWGS in a 12-year-old Caucasian girl with a pituitary macroadenoma and bilateral ovarian masses mimicking a tumour.^3^ Lack of thyroid hormonal feedback leads to thyrotrophic hyperplasia, causing pituitary hyperplasia. This pituitary hyperplasia can be confused with a macroadenoma, which is reported in Case 1. No neurosurgical intervention is needed, as the thyrotrophic hyperplasia subsides with levothyroxine therapy. Children with hypothyroidism have a slow growth rate and increased weight.^7^ Moderate to severe obesity is commonly seen in such individuals, though it is not typical of the disease.^7^ In contrast, our patients were markedly underweight, most likely related to their poor diet due to their lower socioeconomic backgrounds and syndromic diagnoses.

Due to its rare presentation, patients with VWGS often comprise a diagnostic dilemma. Pubertal delay is the well-known consequence of prolonged untreated hypothyroidism.^3^ Diagnosis of VWGS is important, since the pubertal precocity can be reversible and a patient’s genetic height potential can be achieved. Based on the present case series, the clinical septet of precocious puberty, no axillary or pubic hair, absence of goitre, short stature, delayed bone age, elevated TSH with low T3 and T4 (primary hypothyroidism), and raised FSH with pre-pubertal levels of LH suggests VWGS. We hope that this case series with prolonged follow-up will encourage practising paediatricians to optimally and promptly manage hypothyroid patients.

## Conclusions

The diagnosis of VWGS requires a high index of suspicion, and a correct diagnosis has important therapeutic implications. The clinical septet of precocious puberty, no axillary or pubic hair, absence of goitre, short stature, delayed bone age, elevated TSH along with decreased T3 and T4, and high FSH with pre-pubertal levels of LH is the diagnostic hallmark of VWGS. Levothyroxine therapy is the mainstay of treatment, and early diagnosis and treatment can prevent short stature and help attain the genetic height potential of patients diagnosed with VWGS.

**Research highlights**

**What is already known:** Van Wyk–Grumbach syndrome is a rare diagnosis in children with precocious puberty. The exact prevalence of this disorder is not known and few case reports are available in the literature. This rare syndrome is a diagnosis of exclusion.

**What this study adds:** It reiterates the importance of thyroid function as a cause for precocious puberty and emphasizes the importance of proper diagnosis and regular follow-up, which is essential for attaining the genetic height potential and providing a good quality of life.

## References

[1] Rastogi A, Bhadada SK, Bhansali A (2011). An unusual presentation of a usual disorder: Van Wyk-Grumbach syndrome.. Indian J Endocrinol Metab..

[2] Sneha LM, Thanasegarapandian K, Paramasivam V (2013). Short stature and an interesting association.. Indian J Hum Genet..

[3] Durbin KL, Diaz-Montes T, Loveless MB (2011). Van Wyk-Grumbach syndrome: An unusual case and review of literature.. J Pediatr Adolesc Gynecol..

[4] Wormsbecker A, Clarson C (2010). Acquired primary hypothyroidism: Vaginal bleeding in a quiet child.. Can Med Assoc J..

[5] Cetinkaya S, Sagsak E, Erdeve S (2014). Premature menarche associated with Hashimoto thyroiditis at 2 years 9 months: Case report.. J Thyroid Disorders Ther..

[6] Baranowski E (2012). Högler W. An unusual presentation of acquired hypothyroidism: The Van Wyk-Grumbach syndrome.. Eur J Endocrinol..

[7] Danforth E, Horton ES, O’Connel M (1979). Dietary-induced alterations in thyroid hormone metabolism during overnutrition.. J Clin Invest..

